# Resident Operative Autonomy and Attending Verbal Feedback Differ by Resident and Attending Gender

**DOI:** 10.1097/AS9.0000000000000256

**Published:** 2023-02-02

**Authors:** Amanda C. Filiberto, Kenneth L. Abbott, Benjamin Shickel, Brian C. George, Amalia L. Cochran, George A. Sarosi, Gilbert R. Upchurch, Tyler J. Loftus

**Affiliations:** From the * Department of Surgery, University of Florida Health, Gainesville, FL; † Department of Medicine, University of Florida Health, Gainesville, FL; ‡ Department of Learning Health Sciences, University of Michigan, Ann Arbor, MI.

**Keywords:** autonomy, feedback, gender, natural language processing, residency, surgery

## Abstract

**Objectives:**

This study tests the null hypotheses that overall sentiment and gendered words in verbal feedback and resident operative autonomy relative to performance are similar for female and male residents.

**Background:**

Female and male surgical residents may experience training differently, affecting the quality of learning and graduated autonomy.

**Methods:**

A longitudinal, observational study using a Society for Improving Medical Professional Learning collaborative dataset describing resident and attending evaluations of resident operative performance and autonomy and recordings of verbal feedback from attendings from surgical procedures performed at 54 US general surgery residency training programs from 2016 to 2021. Overall sentiment, adjectives, and gendered words in verbal feedback were quantified by natural language processing. Resident operative autonomy and performance, as evaluated by attendings, were reported on 5-point ordinal scales. Performance-adjusted autonomy was calculated as autonomy minus performance.

**Results:**

The final dataset included objective assessments and dictated feedback for 2683 surgical procedures. Sentiment scores were higher for female residents (95 [interquartile range (IQR), 4–100] vs 86 [IQR 2–100]; *P* < 0.001). Gendered words were present in a greater proportion of dictations for female residents (29% vs 25%; *P* = 0.04) due to male attendings disproportionately using male-associated words in feedback for female residents (28% vs 23%; *P* = 0.01). Overall, attendings reported that male residents received greater performance-adjusted autonomy compared with female residents (*P* < 0.001).

**Conclusions:**

Sentiment and gendered words in verbal feedback and performance-adjusted operative autonomy differed for female and male general surgery residents. These findings suggest a need to ensure that trainees are given appropriate and equitable operative autonomy and feedback.

## INTRODUCTION

As graduate medical education shifts towards competency-based models, meaningful attending-to-resident feedback and appropriate provision of graduated autonomy are imperative. Female and male residents may experience training differently, affecting the quality of learning and graduated autonomy.^1–4^ Implicit bias may contribute to observed discrepancies in the way female and male residents experience surgical training.^5,6^

It has been suggested that either gender bias in surgical resident evaluation or the way in which female and male residents experience surgical training is responsible for the observation that female trainees have significantly lower attainment of Accreditation Council for Graduate Medical Education milestones for several subcompetencies.^7^ However, data regarding gender differences in the provision of operative autonomy and equity in performance evaluations are conflicting.^8–11^ It also remains unclear whether the content of verbal feedback from attendings to residents differs by resident and attending gender, as has been described for written performance evaluations.^12^

The Society for Improving and Measuring Procedural Learning (SIMPL) is increasingly being used to better understand the progression of intraoperative resident autonomy and performance, offering unique opportunities to systematically assess associations among surgical resident and attending gender, operative performance, and operative autonomy.^13^ This study evaluates differences in narrative feedback and performance assessments recorded in the SIMPL app with the null hypotheses that overall sentiment and gendered words in verbal feedback and resident operative autonomy relative to performance are similar for female and male residents.

## METHODS

### Study Design and Data Source

This longitudinal, observational study used an existing multicenter dataset maintained by the SIMPL Collaborative, which contains detailed information regarding resident and attending surgeon evaluations of a sample of resident’s operative performance and autonomy as well as verbal feedback from attendings to residents. This study includes both objective and subjective evaluations of resident performance and autonomy in the form of validated ordinal scales as well as verbal feedback recorded on mobile devices, as these elements provide context for one another and are presented together to surgical residents on the SIMPL platform to offer a thorough and holistic evaluation. The University of Florida Institutional Review Board approved this study (IRB No. 202101698). The study was performed in accordance with the Strengthening the Reporting of Observational Studies in Epidemiology reporting guidelines, as shown in Supplemental Digital Content 1 (http://links.lww.com/AOSO/A204).

From 2016 to 2021, the SIMPL app (described in detail at: https://www.simpl.org/simpl, accessed January 13, 2022) was used to generate records representing individual surgical procedures performed by residents at 54 general surgery residency training programs. Variables representing each case were trainee postgraduate year and gender (available classifications on the SIMPL app were female, male, not known, or not applicable; the dataset was filtered to include female and male genders), attending gender, description of procedure type (eg, “Excision soft tissue mass, neck,” “Roux-en-Y gastric bypass (laparoscopic)”), debriefing narratives dictated by attending surgeons, and objective assessments of resident performance and autonomy, assessed separately by attending surgeons and by residents. Resident performance was quantified by the validated Performance Scale, consisting of 5 levels: Unprepared/Critical Deficiency, Inexperienced with Procedure, Intermediate Performance, Practice-Ready Performance, and Exceptional Performance.^13,14^ Resident autonomy was quantified by the Zwisch scale, consisting of 4 levels (Show and Tell, Active Help, Passive Help, or Supervision Only). The Zwisch scale has been shown to be a valid and reliable way to differentiate between levels of faculty guidance provided (and its inverse, resident autonomy granted) during an operation.^13,15,16^

The dataset contained information representing 140,420 evaluations submitted by residents, 98,784 evaluations submitted by attendings, and 4313 dictations submitted by attendings. After excluding blank or duplicated dictations (N = 586) and excluding attending evaluations that were missing dictations (N = 61,961) or objective assessments of resident performance or autonomy (ie, the Performance Scale and Zwisch scale, N = 3226), all remaining evaluations and dictations were merged on common case identification numbers. Cases were then excluded if resident gender was “not known” or “not applicable.” To minimize heterogeneity in the study population, cases were also excluded if the trainee was a preliminary resident or fellow or had postgraduate year 6 or greater. The final dataset included 2683 cases with complete debriefing narratives and objective assessments, as shown in Supplement Digital Content 2 (http://links.lww.com/AOSO/A204).

### Natural Language Processing of Dictated Feedback

This study builds on previous work demonstrating that Natural Language Processing (NLP) can accurately classify feedback quality among 3 surgical residency training programs.^17^ Operative performance assessments by faculty were analyzed with NLP techniques, which allowed for the systematic evaluation of word use to understand overall sentiment and use of gendered language in dictated feedback.

Sentiment analysis is a subtask of NLP encompassing the extraction of opinions, evaluations, attitudes, and emotions from written language.^18^ This study applies advanced sentiment analysis techniques using deep learning methods to assess the degree of positivity or negativity in dictated feedback, which offers the potential advantage of greater contextual understanding of natural language compared with classical approaches to sentiment analysis that use linguistic analysis or rule-based phrase matching against predefined positive and negative word lists.^19^ Pre-trained language models, in which machine learning models are trained on large corpora of generalized human language (eg, the entirety of Wikipedia) and then fine-tuned on a domain and task-specific dataset, have outperformed classical rule-based models for several NLP tasks.^20–22^ The sentiment analysis prediction pipeline was based on a previously established deep learning model that was fine-tuned on the Stanford Sentiment Treebank v2^23^ dataset of movie reviews for predicting positive and negative sentiment from text.^24,25^ Sentiment analysis experiments were conducted using PyTorch and the Huggingface library, truncating dictation transcriptions to 128 tokens (ie, sequences of 1 or more text characters that, when grouped, convey meaning, and represent the smallest unit for processing—in the present study, most tokens were words).^26^ For each transcription, the deep learning model predicted a sentiment label (positive or negative) and a corresponding sentiment score ranging from 0.5 to 1.0. For dictations predicted to be negative, sentiment scores were subtracted from 1.0 so that all transcription scores would range from 0 (most negative) to 100 (most positive). For example, a dictation with positive sentiment and a sentiment score of 0.7 would have a final score of 0.7; a dictation with negative sentiment and a sentiment score of 0.7 would have a final score of 0.3. Examples of positive and negative sentiment in attending verbal dictations are shown in Supplemental Digital Content 3 (http://links.lww.com/AOSO/A204).

This study also classifies gendered words, including both agentic (male-associated) and communal (female-associated) words, adapted from a previously established classification system that has been cited in peer-reviewed literature more than 400 times,^27–31^ as listed in Supplemental Digital Content 4 (http://links.lww.com/AOSO/A204). Each dictation was analyzed using this word bank to determine whether a dictation contained any agentic, communal, or gendered words as well as the proportion of adjectives that were agentic, communal, or gendered words relative to the number of adjectives in the dictation. Although gendered words may be adjectives or nouns, in dictated feedback almost all gendered words were used as adjectives.

### Statistical Analysis

NLP and statistical analyses were performed with Python 3.8.8 software. Discrete variables were compared by Fisher exact test and presented as raw number with percentages. Continuous variables were compared by the Kruskal-Wallis test and presented as median values with interquartile ranges (IQRs). All statistical tests were 2-sided with alpha = 0.05.

Comparisons of cases performed by female versus male residents were made for case characteristics, resident postgraduate year, total number of evaluations per resident for the index case (if the case was a laparoscopic cholecystectomy and the resident had 2 prior evaluations for laparoscopic cholecystectomy then this number would be 3) and for all types of cases (if the resident had 15 total prior evaluations then this number would be 16), and resident and attending assessments of case complexity, resident performance, and resident autonomy. The proportion of gendered words in dictated feedback were compared for female versus male residents with subgroup analyses of dictations by female versus male attendings.

To test the hypothesis that resident operative autonomy relative to performance is similar for female and male residents, it was first necessary to generate a statistical representation of the balance between autonomy and performance for each individual case. This was performed by converting the Performance Scale and Zwisch scale to ordinal scales with maximum score 5 (Performance Scale: exceptional = 5, practice-ready = 4, intermediate = 3, inexperienced = 2, critical deficiency = 1; Zwisch scale: supervision only = 5, passive help = 4, active help = 3, show & tell = 2), and subtracting the performance score from the autonomy score; these scores were compared between female and male residents. Additional analysis included subgroup analyses of dictations by female versus male attendings.

Due to the observation that male residents performed a greater proportion of cases in the “Hardest 1/3” category of case complexity, subgroup analyses excluded cases in the “Hardest 1/3” category, as shown in Supplemental Digital Content 5 and 6 (http://links.lww.com/AOSO/A204).

## RESULTS

### Resident, Attending, and Case Characteristics

Forty-four percent of cases were performed by female residents (N = 1166; Table 1). Seventy-seven percent of cases were performed by residents at postgraduate year 3 or higher. Postgraduate year levels were similar for female and male residents. The total number of all previous evaluations per resident was higher for male residents (18 [IQR 7–14] vs 16 [IQR 7–33]; *P* = 0.005). The number of previous evaluations for the case being evaluated was one (IQR 1–2) for both female and male residents (*P* = 0.80). SIMPL evaluations could be initiated by either residents or attendings; most evaluations were initiated by residents, and a greater proportion of evaluations were initiated by male residents (61% vs 57%; *P* = 0.04).

**TABLE 1.** Resident, Attending, and Case Characteristics

Characteristics, n (%)	Female Residents (n = 1166)	Male Residents (n = 1517)	*P*
Resident postgraduate year			
1	123 (10.5)	132 (8.7)	0.11
2	147 (12.6)	194 (12.8)	0.91
3	288 (24.7)	375 (24.7)	>0.99
4	275 (23.6)	388 (25.6)	0.24
5	333 (28.6)	428 (28.2)	0.86
Total number of evaluations per resident			
For the index case, median (IQR)	1.0 (1.0–2.0)	1.0 (1.0–2.0)	0.80
For all cases, median (IQR)	16.0 (7.0–32.8)	18.0 (7.0–40.0)	**0.005**
Evaluation was initiated by the resident	660 (56.6)	918 (60.5)	**0.04**
Faculty rater gender			
Female	208 (17.8)	290 (19.1)	0.42
Male	958 (82.2)	1227 (80.9)	
Top 20 most common procedures			
Cholecystectomy (laparoscopic)	137 (11.7)	179 (11.8)	>0.99
Inguinal hernia repair (open)	114 (9.8)	129 (8.5)	0.28
Appendectomy (laparoscopic)	73 (6.3)	83 (5.5)	0.41
Ventral hernia repair (open)	44 (3.8)	43 (2.8)	0.19
Inguinal hernia repair (laparoscopic)	28 (2.4)	45 (3.0)	0.40
Cholecystectomy with IOC (laparoscopic)	29 (2.5)	29 (1.9)	0.35
Exploratory laparotomy	22 (1.9)	35 (2.3)	0.50
Ventral hernia repair (laparoscopic)	23 (2.0)	31 (2.0)	>0.99
Umbilical hernia repair (open)	21 (1.8)	31 (2.0)	0.66
Partial colectomy with anastomosis (laparoscopic)	18 (1.5)	28 (1.8)	0.65
Whipple	18 (1.5)	23 (1.5)	>0.99
Mastectomy, partial	12 (1.0)	27 (1.8)	0.14
Small bowel resection with anastomosis (open)	18 (1.5)	14 (0.9)	0.15
Hiatal hernia repair ± fundoplication	6 (0.5)	25 (1.6)	**0.006**
Colostomy/ileostomy takedown (open)	12 (1.0)	17 (1.1)	0.85
Renal transplant without recipient nephrectomy	11 (0.9)	17 (1.1)	0.71
Breast biopsy or excision ± needle localization	18 (1.5)	10 (0.7)	**0.03**
Hepatectomy (open)	15 (1.3)	13 (0.9)	0.34
Partial colectomy with anastomosis (open)	12 (1.0)	13 (0.9)	0.69
Orthotopic liver transplant	11 (0.9)	13 (0.9)	0.84
Resident assessment of case complexity			
Easiest 1/3	163 (14.0)	192 (12.7)	0.33
Average	686 (58.8)	849 (56.0)	0.15
Hardest 1/3	136 (11.7)	227 (15.0)	**0.01**
Missing	181 (15.5)	249 (16.4)	0.56
Attending assessment of case complexity			
Easiest 1/3	199 (17.1)	252 (16.6)	0.76
Average	690 (59.2)	854 (56.3)	0.15
Hardest 1/3	277 (23.8)	411 (27.1)	**0.05**
Missing	0 (0.0)	0 (0.0)	>0.99

*P* ≤ 0.05.

IOC indicates intraoperative cholangiogram.

The top 3 most common procedures were laparoscopic cholecystectomy, open inguinal hernia repair, and laparoscopic appendectomy. For 18 of the top 20 most common procedures, there were similar proportions of cases performed by female and male residents. Male residents performed a greater proportion of hiatal hernia repairs (1.6% vs 0.5%; *P* = 0.006); female residents performed a greater proportion of breast excisional biopsies (1.5% vs 0.7%; *P* = 0.03). Greater proportions of cases classified as “Hardest 1/3” were performed by male residents as compared with female residents, according to both resident (15% vs 12%; *P* = 0.01) and attending (27% vs 24%; *P* = 0.05) assessments of case complexity, as shown in Table 1. Case characteristics for a subset of “Easiest 1/3” and “Average” complexity cases were otherwise similar to case characteristics in the primary analysis, as summarized in Supplemental Digital Content 5 (http://links.lww.com/AOSO/A204).

### Operative Performance, Autonomy, Sentiment, and Gendered Words in Dictated Feedback

A greater proportion of male residents assessed their own performance as “intermediate” (52%, N = 793 vs 46%, N = 537; *P* = 0.001); a greater proportion of female residents assessed their own performance as “practice-ready” (18%, N = 213 vs 13%, N = 197; *P* < 0.001; Table 2). Attendings evaluated a greater proportion of male residents as “inexperienced with procedure” (18%, N = 265 vs 12%, N = 141; *P* = 0.001). A greater proportion of female residents assessed their operative autonomy as “supervision only” (10%, N = 116 vs 6%, N = 97; *P* < 0.001). Attending assessments of resident autonomy were similar between female and male residents. Similar associations among resident performance, autonomy, and gender were observed when excluding “Hardest 1/3” cases (Supplemental Digital Content 7, http://links.lww.com/AOSO/A204).

**TABLE 2.** Resident and Attending Assessments of Resident Operative Performance and Autonomy and the Use of Gendered Words in Dictated Feedback

Evaluation Results, n (%)	Female Residents (n = 1166)	Male Residents (n = 1517)	*P*
Resident assessment of resident performance			
Critical deficiency	1 (0.1)	1 (0.1)	>0.99
Inexperienced with procedure	161 (13.8)	183 (12.1)	0.18
Intermediate	537 (46.1)	793 (52.3)	**0.001**
Practice-ready	213 (18.3)	197 (13.0)	**<0.001**
Exceptional	6 (0.5)	2 (0.1)	0.09
Missing	248 (21.3)	341 (22.5)	0.48
Attending assessment of resident performance			
Critical deficiency	2 (0.2)	0 (0.0)	0.19
Inexperienced with procedure	141 (12.1)	265 (17.5)	**<0.001**
Intermediate	621 (53.3)	778 (51.3)	0.31
Practice-ready	364 (31.2)	428 (28.2)	0.10
Exceptional	38 (3.3)	46 (3.0)	0.74
Missing	0 (0.0)	0 (0.0)	>0.99
Resident assessment of resident autonomy			
Show & tell	67 (5.7)	92 (6.1)	0.74
Active help	522 (44.8)	708 (46.7)	0.33
Passive help	280 (24.0)	371 (24.5)	0.82
Supervision only	116 (9.9)	97 (6.4)	**<0.001**
Missing	181 (15.5)	249 (16.4)	0.56
Attending assessment of resident autonomy			
Show & tell	0 (0.0)	0 (0.0)	>0.99
Active help	640 (54.9)	835 (55.0)	0.94
Passive help	380 (32.6)	507 (33.4)	0.68
Supervision only	146 (12.5)	175 (11.5)	0.44
Missing	0 (0.0)	0 (0.0)	>0.99
Feedback from attendings to residents, per dictation			
Sentiment score, median (IQR)	94.7 (4.1–99.7)	86.2 (2.3–99.5)	**<0.001**
Overall sentiment was positive, n (%)	722 (61.9)	867 (57.2)	**0.01**
Number of words, median (IQR)	108.0 (61.0–182.8)	109.0 (64.0–192.0)	0.22
Number of adjectives, median (IQR)	11.0 (6.0–17.0)	11.0 (7.0–18.0)	0.23
Communal word(s) present in dictation, n (%)	20 (1.7)	37 (2.4)	0.23
Proportion* of communal words, median (IQR)	0.0 (0.0–0.0)	0.0 (0.0–0.0)	0.195
Agentic word(s) present in dictation, n (%)	323 (27.7)	352 (23.2)	**0.01**
Proportion* of agentic words, median (IQR)	0.0 (0.0–3.8)	0.0 (0.0–0.0)	**0.008**
Gendered word(s) present in dictation, n (%)	334 (28.6)	381 (25.1)	**0.04**
Proportion* of gendered words, median (IQR)	0.0 (0.0–4.6)	0.0 (0.0–1.9)	**0.03**

*P* ≤ 0.05.

*Proportions are relative to the number adjectives in the dictation.

Female residents had higher sentiment scores (95 [IQR 4–100] vs 86 [IQR 2–100]; *P* < 0.001). A greater proportion of cases performed by female residents had an overall positive sentiment (62% vs 57%; *P* = 0.01). There were no gender differences in the total number of words, adjectives, or communal words used in dictated feedback. Gendered words were present in a greater proportion of dictations for female residents (29% vs 25%; *P* = 0.04); this difference was attributable to male attendings using agentic (male-associated) words in a greater proportion of dictations for female residents (28% vs 23%; *P* = 0.01). Overall, the number of gendered words divided by number of adjectives was greater for female residents (0, IQR 0–5 vs 0, IQR 0–2; *P* = 0.03), as illustrated in Supplemental Digital Content 8 (http://links.lww.com/AOSO/A204). For cases by female attendings, gendered words represented less than 25% of all adjectives for both female and male residents, such that the distributions are not visible in the boxplot figure. For cases by male attendings, the proportion of gendered words in dictations was greater for female residents (0, IQR 0–5 vs 0, IQR 0–2; *P* = 0.02).

Characteristics and results from subgroup analyses for cases by male attendings only and cases by female attendings only are summarized in Supplemental Digital Content 9–12 (http://links.lww.com/AOSO/A204). For cases by male attendings, objective assessments of resident performance and autonomy mirrored the primary analysis, sentiment scores were higher for female residents (90 [IQR 3–100] vs 79 [IQR 2–99]; *P* = 0.02) but proportions of cases with overall positive sentiment were similar for female and male residents. For cases by female attendings, operative performance and autonomy were similar for female and male residents, sentiment scores were higher for females (99 [IQR 52–100] vs 96 [IQR 4–100]; *P* < 0.001), and proportions of cases with overall positive sentiment were higher for female residents (75% vs 63%; *P* = 0.005).

**FIGURE 1.**
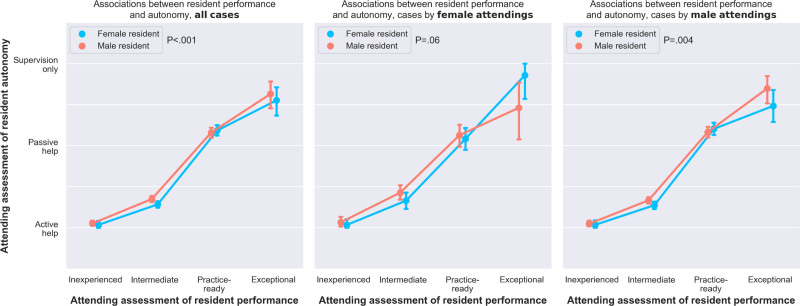
Performance-adjusted autonomy for female and male residents.

Associations between attending assessments of resident autonomy and resident performance are illustrated in Figure 1. When considering all cases performed by both female and male attendings, progressive increases in resident performance were associated with progressive increases in resident autonomy. Overall, male residents had greater autonomy relative to performance compared with female residents, as determined by subtracting the ordinal performance score for each case from the ordinal autonomy score for the same case and comparing aggregate results by resident gender (*P* < 0.001). Male residents had greater autonomy relative to performance compared with female residents when the attending was male (*P* = 0.004) and when the attending was female, although the difference for female attendings was not statistically significant (*P* = 0.06). When high-complexity cases were excluded, again, male residents had greater autonomy relative to performance compared with female residents (*P* = 0.004); this was true whether the attending was female (*P* = 0.04) or male (*P* = 0.03; Supplemental Digital Content 6, http://links.lww.com/AOSO/A204). There were no significant differences in autonomy relative to performance at individual performance levels, as suggested by overlapping 95% confidence intervals.

## DISCUSSION

These findings suggest sentiment and gendered words in dictated feedback from attendings to residents and alignment between resident operative performance and autonomy may be different for female and male residents. Overall, attendings reported that male residents were granted greater autonomy relative to their operative performance compared with female residents. Surprisingly, the increased use of gendered words in dictated feedback for female residents was due to greater use of agentic (traditionally male-associated) words. Female residents had higher sentiment scores and overall sentiment positivity compared with male residents, which was more evident in female attending verbal feedback. These findings occurred in the absence of differences in resident postgraduate year or case complexity. There were subtle differences in the types of cases performed by female and male residents, but these cases represented a small proportion of the dataset. Male residents had more total prior evaluations for cases of any type, although the authors are unaware of any evidence that the total number of cases performed by graduating residents differs between female and male residents.

A recent study used NLP to detect linguistic differences and gender bias in letters of recommendation written for general surgery residency applicants and found that letters of recommendation for females contained more gendered wording compared with letters for males, and that female applicants who were described with male-associated words had higher sentiment scores, consistent with the present study.^29^ When female attending verbal dictations were evaluated herein, the use of gendered words was similar for female and male residents but sentiment score and overall positive sentiment were higher for female residents, suggesting that female attendings provided comparatively gender-neutral verbal feedback but were verbally more positive for female residents. The available data do not allow for understanding of why female attendings provided comparatively gender-neutral feedback but with greater positivity for female residents. These findings could reflect a progression towards gender equity in surgery and “lift as you climb” movements such as online campaigns put forth by the Association of Women in Surgery to connect female surgeons with one another.^32^ Importantly, while positive feedback and encouragement may serve to build confidence and reinforce good habits, negative feedback is occasionally necessary for growth, and should be valued by residents and attendings alike, regardless of gender. Too much positivity, unanchored to negativity and sober recognition of challenges and opportunities for growth, has been associated with lower business performance.^33,34^ Maintaining optimal levels of positivity (not too much, not too little) has the potential to cultivate grit and improve human performance for complex tasks like surgery, suggesting that positivity in dictated feedback is relevant to the quest of optimizing surgical training.^33^ Overall, our findings suggest that gender differences exist in the form of verbal feedback from female attendings, and underscore the importance of ensuring that trainees are given appropriate and equitable operative autonomy and feedback.

Few prior studies have assessed associations between trainee gender and operative autonomy, and none report autonomy relative to performance.^8,9,35–37^ Three studies of thoracic and general surgery training programs similarly found that female trainees were granted less operative autonomy compared with male counterparts.^8,9,37^ However, in studies using a different measurement tool to assess entrustment in the operating room, using observation and assessment by a third party, there was no association between resident gender and resident autonomy granted by faculty.^35,36^ The latter studies may have been influenced by the Hawthorne effect, which could account for differences in results relative to the former studies (ie, provision of autonomy was equitable while provision of autonomy was being observed and recorded).

In 2018, the American Board of Surgery initiated the Entrustable Professional Activities (EPA) pilot project, “designed to integrate competencies and milestones to provide an evaluation platform that translates often-theoretical concepts into a tool that can be used in a single patient encounter.”^38^ In a recent study evaluating the effect of general surgery resident gender on EPA entrustment levels, Padilla et al^39^ used NLP methods to analyze narrative comments in identifying topics correlated with resident sex. Faculty assessments showed no differences in EPA levels between female and male residents. Additionally, resident self-ratings were lower for female residents compared with male residents, which the authors describe a theoretical framework for perceived differences in autonomy. This important work showed that EPA-based evaluation, when performed by attendings, can be gender neutral, which is a powerful argument for moving surgical training toward competency-based assessment models fueled by frequent workplace-based assessment.^40^

Progressive autonomy in the operating room is a useful measure of preparedness for independent practice and will remain an important assessment tool even during the planned implementation of competency-based education. This study was not designed to assess underlying causes of the observed differences in autonomy relative to performance seen between male and female residents. It is, however, important that trainees be given adequate operative autonomy throughout their training that should not differ by gender or other sociodemographic factors. One way to mitigate bias in autonomy may be to specifically articulate which procedure-specific behaviors should be displayed for residents to gain graduated levels of autonomy. Additionally, it is imperative for educators to acknowledge that there is potential room for subjectivity and bias within assessment tools and that to create a work environment in which all trainees can succeed, these biases must be acknowledged and mitigated. For SIMPL, this could be accomplished by expanding the existing guidance for providing verbal feedback, which is currently provided to attendings in training sessions and within the mobile application.

### Limitations

The determination of gender was based on a binary response option as designed by the SIMPL application, and although this may be a gross representation of the breakdown of male and female residents in a program, it may not be representative of transgender and gender nonconforming individuals. Further, data collected using the SIMPL app represents a small portion of observed clinical performances, and trainees may disproportionately solicit feedback for cases in which they performed well or are interested in improving upon. Therefore, the cases included in this study may not truly be representative of the full breadth of experience of surgical trainees. A recent study using SIMPL data confirmed that there was a strong correlation between SIMPL procedure frequencies and Accreditation Council for Graduate Medical Education case log procedure frequencies.^41^ The present study only includes cases logged by attendings and residents if attending verbal dictations were provided, which may generate reporting bias. The present study included the same top 3 most common procedures (laparoscopic cholecystectomy, laparoscopic appendectomy, and open inguinal hernia repair) with similar procedure frequencies; however, there were substantial differences in the remaining procedures included in the study, likely attributable to the factors described above. Sentiment in dictations was assessed using a publicly available benchmark model; a domain-specific training dataset may lead to more accurate sentiment predictions.

## CONCLUSIONS

Gendered words, especially male-associated words, were present in a greater proportion of dictations for female trainees compared with male trainees, primarily due to male attendings using male-associated words in feedback for female residents. Female residents received higher sentiment scores while male residents received greater performance-adjusted autonomy. These findings suggest the need to ensure that trainees are given appropriate and equitable operative autonomy and feedback that should not differ by gender or other sociodemographic factors. To promote equity in resident operative autonomy, we propose that attendings articulate procedure-specific behaviors that should be displayed for residents to gain graduated levels of autonomy. To promote equity in verbal feedback, we propose the implementation of specific guidance for the verbal feedback portion of SIMPL evaluations. For individual attendings, we suggest granting residents autonomy that corresponds to their operative performance and providing equitable, formative feedback that builds better surgeons, regardless of gender.

## Supplementary Material



## References

[1] Girod S Fassiotto M Grewal D. Reducing implicit gender leadership bias in academic medicine with an educational intervention. Acad Med. 2016;91:1143–1150. 26826068 10.1097/ACM.0000000000001099

[2] Hansen M Schoonover A Skarica B. Implicit gender bias among US resident physicians. BMC Med Educ. 2019;19:396. 31660944 10.1186/s12909-019-1818-1 PMC6819402

[3] Hui K Sukhera J Vigod S. Recognizing and addressing implicit gender bias in medicine. CMAJ. 2020;192:E1269–E1270. 33077523 10.1503/cmaj.200286 PMC7588202

[4] Mueller AS Jenkins TM Osborne M. Gender differences in attending physicians’ feedback to residents: a qualitative analysis. J Grad Med Educ. 2017;9:577–585. 29075375 10.4300/JGME-D-17-00126.1 PMC5646913

[5] Hemphill ME Maher Z Ross HM. Addressing gender-related implicit bias in surgical resident physician education: a set of guidelines. J Surg Educ. 2020;77:491–494. 31954662 10.1016/j.jsurg.2019.12.014

[6] Ali A Subhi Y Ringsted C. Gender differences in the acquisition of surgical skills: a systematic review. Surg Endosc. 2015;29:3065–3073. 25631116 10.1007/s00464-015-4092-2

[7] Landau SI Syvyk S Wirtalla C. Trainee sex and accreditation council for graduate medical education milestone assessments during general surgery residency. JAMA Surg. 2021;156:925–931. 34232269 10.1001/jamasurg.2021.3005 PMC8264749

[8] Meyerson SL Odell DD Zwischenberger JB. The effect of gender on operative autonomy in general surgery residents. Surgery. 2019;166:738–743. 31326184 10.1016/j.surg.2019.06.006 PMC7382913

[9] Joh DB van der Werf B Watson BJ. Assessment of autonomy in operative procedures among female and male New Zealand general surgery trainees. JAMA Surg. 2020;155:1019–1026. 32857160 10.1001/jamasurg.2020.3021 PMC7450402

[10] Lane SM Young KA Hayek SA. Meaningful autonomy in general surgery training: exploring for gender bias. Am J Surg. 2020;219:240–244. 31801653 10.1016/j.amjsurg.2019.11.035

[11] Cookenmaster C Shebrain S Vos D. Gender perception bias of operative autonomy evaluations among residents and faculty in general surgery training. Am J Surg. 2021;221:515–520. 33189312 10.1016/j.amjsurg.2020.11.016

[12] Gerull KM Loe M Seiler K. Assessing gender bias in qualitative evaluations of surgical residents. Am J Surg. 2019;217:306–313. 30343879 10.1016/j.amjsurg.2018.09.029 PMC8687875

[13] Bohnen JD George BC Williams RG. The feasibility of real-time intraoperative performance assessment with SIMPL (System for Improving and Measuring Procedural Learning): early experience from a multi-institutional trial. J Surg Educ. 2016;73:e118–e130. 27886971 10.1016/j.jsurg.2016.08.010

[14] Williams RG Sanfey H Chen XP. A controlled study to determine measurement conditions necessary for a reliable and valid operative performance assessment: a controlled prospective observational study. Ann Surg. 2012;256:177–187. 22751518 10.1097/SLA.0b013e31825b6de4

[15] DaRosa DA Zwischenberger JB Meyerson SL. A theory-based model for teaching and assessing residents in the operating room. J Surg Educ. 2013;70:24–30. 23337666 10.1016/j.jsurg.2012.07.007

[16] George BC Teitelbaum EN Meyerson SL. Reliability, validity, and feasibility of the Zwisch scale for the assessment of intraoperative performance. J Surg Educ. 2014;71:e90–e96. 25192794 10.1016/j.jsurg.2014.06.018

[17] Otles E Kendrick D Solano QP. Using natural language processing to automatically assess feedback quality: findings from three surgical residencies. Acad Med. 2021;96:1457–1460. 33951682 10.1097/ACM.0000000000004153

[18] Liu B. Sentiment Analysis and Opinion Mining: Morgan & Claypool. Morgan & Claypool Publishers; 2012.

[19] Vaswani A Shazeer N Parmar N. Attention is all you need. Adv Neural Inf Process Systems. 2017;30.

[20] Qiu XP Sun TX Xu YG. Pre-trained models for natural language processing: a survey. Sci China-Technol Sci. 2020;63:1872–1897.

[21] Brown T Mann B Ryder N. Language models are few-shot learners. Adv Neural Inf Process Systems. 2020;33:1877–1901.

[22] Devlin J Chang MW Lee K. Bert: pre-training of deep bidirectional transformers for language understanding. arXiv. Preprint posted online October 11, 2018. doi: 10.48550/arXiv.1810.04805.

[23] Socher R Perelygin A Wu J. Recursive deep models for semantic compositionality over a sentiment treebank. Proceedings of the 2013 Conference on Empirical Methods in Natural Language Processing. October 18-21, 2013; Seattle, WA, pp. 1631–1642.

[24] Sanh V Debut L Chaumond J. DistilBERT, a distilled version of BERT: smaller, faster, cheaper and lighter. arXiv. Preprint posted online October 2, 2019. doi: 10.48550/arXiv.1910.01108.

[25] Hinton G Vinyals O Dean J. Distilling the knowledge in a neural network. arXiv. Preprint posted online March 9, 2015. doi: 10.48550/arXiv.1503.02531.

[26] Wolf T Debut L Sanh V. Huggingface’s transformers: state-of-the-art natural language processing. arXiv. Preprint posted online October 9, 2019. doi: 10.48550/arXiv.1910.03771.

[27] Madera JM Hebl MR Martin RC. Gender and letters of recommendation for academia: agentic and communal differences. J Appl Psychol. 2009;94:1591–1599. 19916666 10.1037/a0016539

[28] Khan S Kirubarajan A Shamsheri T. Gender bias in reference letters for residency and academic medicine: a systematic review [published online ahead of print June 2, 2021]. Postgrad Med J. doi: 10.1136/postgradmedj-2021-140045. 10.1136/postgradmedj-2021-140045 37222712

[29] Sarraf D Vasiliu V Imberman B. Use of artificial intelligence for gender bias analysis in letters of recommendation for general surgery residency candidates. Am J Surg. 2021;222:1051–1059. 34674847 10.1016/j.amjsurg.2021.09.034

[30] Sheffield V Hartley S Stansfield RB. Gendered expectations: the impact of gender, evaluation language, and clinical setting on resident trainee assessment of faculty performance. J Gen Intern Med. 2022;37:714–722. 10.1007/s11606-021-07093-w PMC8904706 34405349

[31] Truong J Santarelli A Dawson A. Gender differences in language of standardized letter of evaluation narratives in osteopathic emergency medicine residency applicants. Cureus. 2021;13:e16622. 34458034 10.7759/cureus.16622 PMC8384531

[32] Lim WH Wong C Jain SR. The unspoken reality of gender bias in surgery: a qualitative systematic review. PLoS One. 2021;16:e0246420. 33529257 10.1371/journal.pone.0246420 PMC7853521

[33] Loftus TJ Filiberto AC Rosenthal MD. Performance advantages for grit and optimism. Am J Surg. 2020;220:10–18. 32098653 10.1016/j.amjsurg.2020.01.057

[34] Losada M Heaphy E. The role of positivity and connectivity in the performance of business teams - a nonlinear dynamics model. Am Behav Sci. 2004;47:740–765.

[35] Nikolian VC Sutzko DC Georgoff PE. Improving the feasibility and utility of OpTrust-a tool assessing intraoperative entrustment. Am J Surg. 2018;216:13–18. 29128100 10.1016/j.amjsurg.2017.10.036

[36] Thompson-Burdine J Sutzko DC Nikolian VC. Impact of a resident’s sex on intraoperative entrustment of surgery trainees. Surgery. 2018;164:583–588. 30041964 10.1016/j.surg.2018.05.014

[37] Meyerson SL Sternbach JM Zwischenberger JB. The effect of gender on resident autonomy in the operating room. J Surg Educ. 2017;74:e111–e118. 28669788 10.1016/j.jsurg.2017.06.014

[38] Brazelle M Zmijewski P McLeod C. Concurrent validity evidence for entrustable professional activities in general surgery residents. J Am Coll Surg. 2022;234:938–946. 35426408 10.1097/XCS.0000000000000168

[39] Padilla EP Stahl CC Jung SA. Gender differences in entrustable professional activity evaluations of general surgery residents. Ann Surg. 2022;275:222–229. 33856381 10.1097/SLA.0000000000004905 PMC8514571

[40] Sarosi GA Jr Klingensmith M. Entrustable professional activities, a tool for addressing sex bias and the imposter syndrome? Ann Surg. 2022;275:230–231. 34433185 10.1097/SLA.0000000000005189

[41] Abbott KL Krumm AE Clark MJ. Representativeness of workplace-based operative performance assessments for resident operative experience. J Surg Educ. 2022;79:769–774. 34996745 10.1016/j.jsurg.2021.12.010

