# Supplementary material for: Resident Operative Autonomy and Attending Verbal Feedback Differ by Resident and Attending Gender
Source: Ann Surg Open. 2023 Feb 2;4(1):e256. doi: 10.1097/AS9.0000000000000256 (PMC10431433; doi:10.1097/AS9.0000000000000256)
Supplement: Supplementary file 1 [file as9-4-e256-s001.pdf]

Supplemental Digital Content 1. STROBE Statement Checklist for Cohort Study

	Item No	Recommendation	Page No
**Title and abstract**	1	(*a*) Indicate the study's design with a commonly used term in the title or the abstract<br>(*b*) Provide in the abstract an informative and balanced summary of what was done and what was found	3
**Introduction**			
Background/rationale	2	Explain the scientific background and rationale for the investigation being reported	5
Objectives	3	State specific objectives, including any prespecified hypotheses	5-6
**Methods**			
Study design	4	Present key elements of study design early in the paper	6
Setting	5	Describe the setting, locations, and relevant dates, including periods of recruitment, exposure, follow-up, and data collection	6
Participants	6	(*a*) Give the eligibility criteria, and the sources and methods of selection of participants. Describe methods of follow-up<br>(*b*) For matched studies, give matching criteria and number of exposed and unexposed	6
Variables	7	Clearly define all outcomes, exposures, predictors, potential confounders, and effect modifiers. Give diagnostic criteria, if applicable	6
Data sources/ measurement	8*	For each variable of interest, give sources of data and details of methods of assessment (measurement). Describe comparability of assessment methods if there is more than one group	6
Bias	9	Describe any efforts to address potential sources of bias	
Study size	10	Explain how the study size was arrived at	7
Quantitative variables	11	Explain how quantitative variables were handled in the analyses. If applicable, describe which groupings were chosen and why	9
Statistical methods	12	(*a*) Describe all statistical methods, including those used to control for confounding<br>(*b*) Describe any methods used to examine subgroups and interactions<br>(*c*) Explain how missing data were addressed<br>(*d*) If applicable, explain how loss to follow-up was addressed<br>(*e*) Describe any sensitivity analyses	9-10
**Results**			
Participants	13*	(a) Report numbers of individuals at each stage of study—eg numbers potentially eligible, examined for eligibility, confirmed eligible, included in the study, completing follow-up, and analysed<br>(b) Give reasons for non-participation at each stage<br>(c) Consider use of a flow diagram	7
Descriptive data	14*	(a) Give characteristics of study participants (eg demographic, clinical, social) and information on exposures and potential confounders<br>(b) Indicate number of participants with missing data for each variable of interest<br>(c) Summarise follow-up time (eg, average and total amount)	10
Outcome data	15*	Report numbers of outcome events or summary measures over time	10-13
Main results	16	(*a*) Give unadjusted estimates and, if applicable, confounder-adjusted estimates and their precision (eg, 95% confidence interval). Make clear which confounders were adjusted for and why they were included<br>(*b*) Report category boundaries when continuous variables were categorized<br>(*c*) If relevant, consider translating estimates of relative risk into absolute risk for a meaningful time period	10-13
Other analyses	17	Report other analyses done—eg analyses of subgroups and interactions, and sensitivity analyses	10-13

**Discussion**			
Key results	18	Summarise key results with reference to study objectives	13-14
Limitations	19	Discuss limitations of the study, taking into account sources of potential bias or imprecision. Discuss both direction and magnitude of any potential bias	15-16
Interpretation	20	Give a cautious overall interpretation of results considering objectives, limitations, multiplicity of analyses, results from similar studies, and other relevant evidence	16
Generalisability	21	Discuss the generalisability (external validity) of the study results	16
**Other information**			
Funding	22	Give the source of funding and the role of the funders for the present study and, if applicable, for the original study on which the present article is based	17

Supplemental Digital Content 2. Flowchart of case identification and inclusion and exclusion criteria.

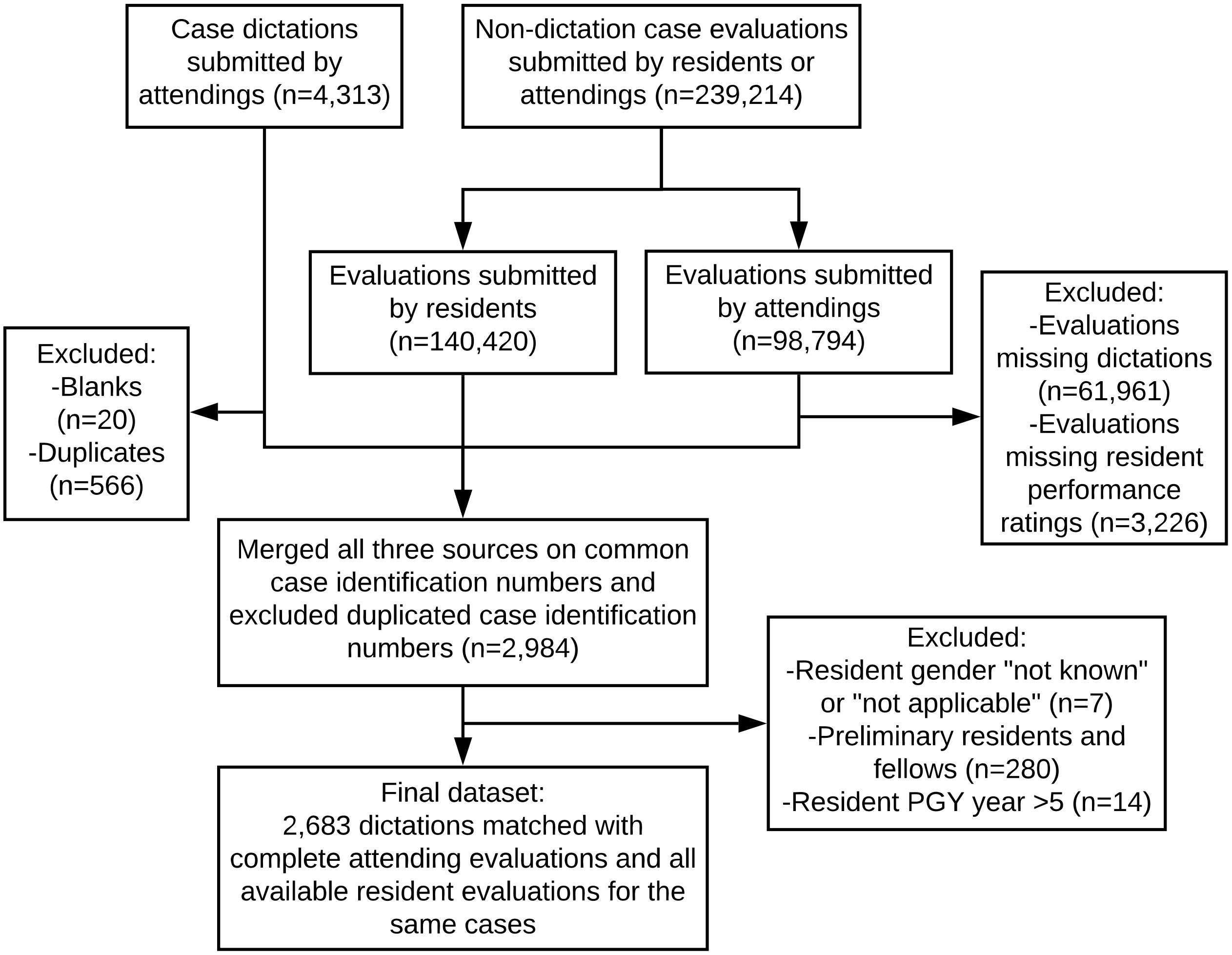

Supplemental Digital Content 3. Sentiment in verbal dictations

Example of negative dictation:	Example of positive dictation:
"Regarding his laparoscopic ability [resident name] needs to improve upon using both hands to be efficient and smooth he seemed to be operating with only one hand his dominant hand while the other hand was not really helping."	"Excellent job with this exploratory laparotomy and repair of duodenal perforation [resident name]'s tissue handling was excellent her overall performance in the operation showed that she understood the steps and she did not waste time she had good confidence and she was able to lead the intern through the critical steps of the operation."

Supplemental Digital Content 4. List of gendered words.

Communal	Agentic
Affectionate	Superb
Sympathetic	Excellent
Nurturing	Outstanding
Warm	Assertive
Thoughtful	Dominant
Delightful	Forceful
Compassionate	Exemplary
Friendly	Confident
Husband/wife/spouse	Leader
Children	Strong
Empathetic	Efficient
Team player	Problem-solver
Easy to work with	Intelligent
Well-liked	Bright
Communication skills	Excel
Conscientious	Exceptional
Honest	Rising star
Humble	Well-rounded
Calm	Bright future
Congenial	

Supplemental Digital Content 5. Resident, attending, and case characteristics for easiest third and average complexity cases.

**Characteristics**, n (%)	**Female residents** (n=854)	**Male residents** (n=1,046)	**P**
**Resident postgraduate year**			
1	108 (12.6)	117 (11.2)	.35
2	112 (13.1)	151 (14.4)	.42
3	216 (25.3)	260 (24.9)	.83
4	198 (23.2)	250 (23.9)	.75
5	220 (25.8)	268 (25.6)	.96
**Total number of evaluations per resident**			
For the index case, median [IQR]	1.0 [1.0-2.0]	1.0 [1.0-2.0]	.31
For all cases, median [IQR]	15.0 [7.0-32.0]	17.0 [7.0-41.0]	**.02**
**Evaluation was initiated by the resident**	478 (56.0)	608 (58.1)	.35
**Faculty rater gender**			
Female	152 (17.8)	206 (19.7)	.32
Male	702 (82.2)	840 (80.3)	
**Top 20 most common procedures**			
Cholecystectomy (laparoscopic)	97 (11.4)	131 (12.5)	.48
Inguinal hernia repair (open)	82 (9.6)	94 (9.0)	.69
Appendectomy (laparoscopic)	48 (5.6)	52 (5.0)	.54
Ventral hernia repair (open)	35 (4.1)	29 (2.8)	.13
Inguinal hernia repair (laparoscopic)	21 (2.5)	33 (3.2)	.41
Cholecystectomy with IOC (laparoscopic)	18 (2.1)	13 (1.2)	.15
Exploratory laparotomy	12 (1.4)	19 (1.8)	.59
Ventral hernia repair (laparoscopic)	17 (2.0)	25 (2.4)	.64
Umbilical hernia repair (open)	18 (2.1)	28 (2.7)	.46
Partial colectomy with anastomosis (laparoscopic)	11 (1.3)	17 (1.6)	.57
Whipple	7 (0.8)	15 (1.4)	.28
Mastectomy, partial	10 (1.2)	24 (2.3)	.08
Small bowel resection with anastomosis (open)	16 (1.9)	9 (0.9)	.07
Hiatal hernia repair +/- fundoplication	6 (0.7)	17 (1.6)	.09
Colostomy/Ileostomy takedown (open)	8 (0.9)	13 (1.2)	.66
Renal transplant without recipient nephrectomy	10 (1.2)	12 (1.1)	>.99
Breast biopsy or excision +/- needle localization	17 (2.0)	9 (0.9)	**.05**
Hepatectomy (open)	9 (1.1)	8 (0.8)	.63
Partial colectomy with anastomosis (open)	10 (1.2)	6 (0.6)	.21
Orthotopic liver transplant	10 (1.2)	7 (0.7)	.33
**Resident assessment of case complexity**			
Easiest 1/3	163 (19.1)	183 (17.5)	.37
Average	553 (64.8)	676 (64.6)	.96
Hardest 1/3	0 (0.0)	0 (0.0)	>.99
Missing	138 (16.2)	187 (17.9)	.33
**Attending assessment of case complexity**			
Easiest 1/3	195 (22.8)	248 (23.7)	.66
Average	659 (77.2)	798 (76.3)	.66
Hardest 1/3	0 (0.0)	0 (0.0)	>.99
Missing	0 (0.0)	0 (0.0)	>.99

Supplemental Digital Content 6. Performance-adjusted autonomy for female and male residents for easiest third and average complexity cases.

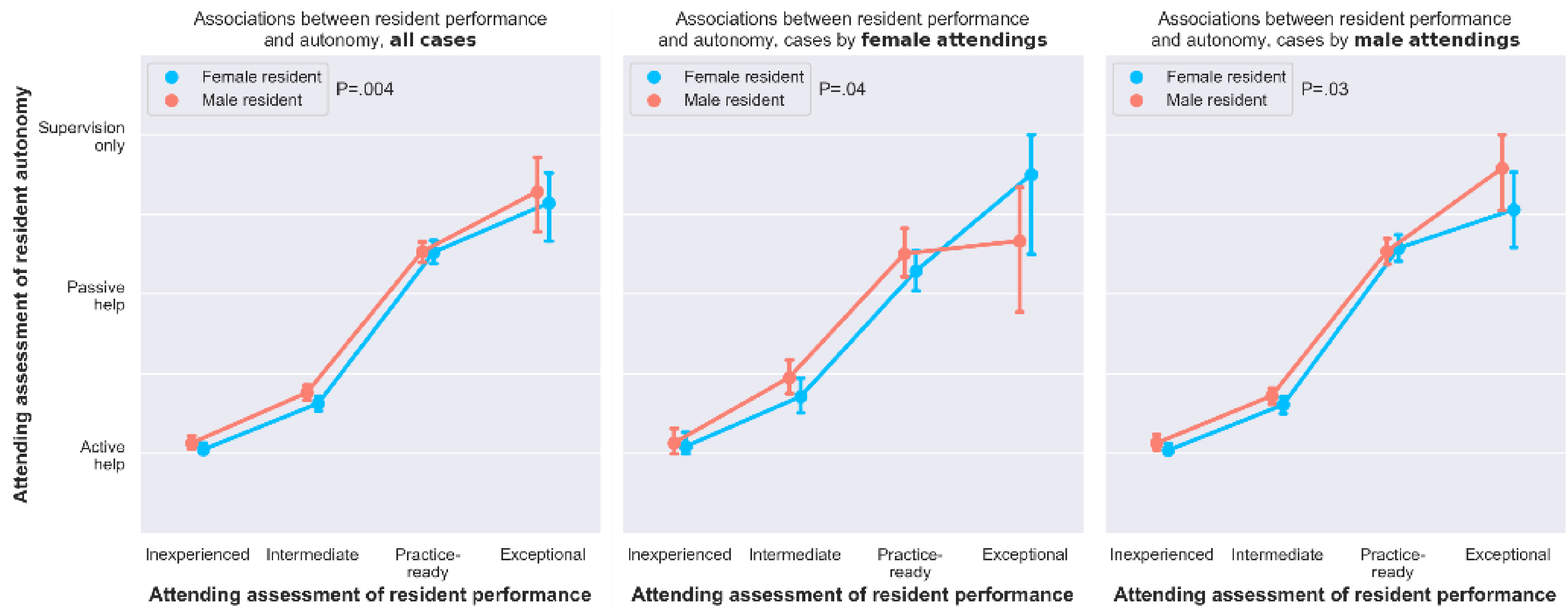

Supplemental Digital Content 7. Resident and attending assessments of resident operative performance and autonomy and the use of gendered words in dictated feedback for easiest third and average complexity cases.

**Evaluation results**, n (%)	**Female residents** (n=1,166)	**Male residents** (n=1,517)	**P**
**Resident assessment of resident performance**			
Critical deficiency	1 (0.1)	1 (0.1)	>.99
Inexperienced with procedure	123 (14.4)	131 (12.5)	.25
Intermediate	388 (45.4)	523 (50.0)	**.05**
Practice-ready	151 (17.7)	144 (13.8)	**.02**
Exceptional	5 (0.6)	1 (0.1)	.10
Missing	186 (21.8)	246 (23.5)	.38
**Attending assessment of resident performance**			
Critical deficiency	2 (0.2)	0 (0.0)	.20
Inexperienced with procedure	118 (13.8)	194 (18.5)	**.006**
Intermediate	443 (51.9)	523 (50.0)	.43
Practice-ready	270 (31.6)	301 (28.8)	.19
Exceptional	21 (2.5)	28 (2.7)	.89
Missing	0 (0.0)	0 (0.0)	>.99
**Resident assessment of resident autonomy**			
Show & tell	48 (5.6)	59 (5.6)	>.99
Active help	370 (43.3)	465 (44.5)	.64
Passive help	202 (23.7)	253 (24.2)	.83
Supervision only	96 (11.2)	82 (7.8)	**.01**
Missing	138 (16.2)	187 (17.9)	.33
**Attending assessment of resident autonomy**			
Show & tell	0 (0.0)	0 (0.0)	>.99
Active help	452 (52.9)	547 (52.3)	.82
Passive help	287 (33.6)	358 (34.2)	.81
Supervision only	115 (13.5)	141 (13.5)	>.99
Missing	0 (0.0)	0 (0.0)	>.99
**Feedback from attendings to residents, per dictation**			
Sentiment score, median [IQR]	94.8 [4.0-99.7]	84.8 [2.2-99.4]	**.001**
Overall sentiment was positive, n (%)	530 (62.1)	599 (57.3)	**.04**
Number of words, median [IQR]	104.5 [59.0-179.0]	111.0 [63.0-192.0]	.08
Number of adjectives, median [IQR]	10.0 [6.0-17.0]	11.0 [7.0-18.0]	.06
Communal word(s) present in dictation, n (%)	15 (1.8)	23 (2.2)	.52
Proportion[a] of communal words, median [IQR]	0.0 [0.0-0.0]	0.0 [0.0-0.0]	.49
Agentic word(s) present in dictation, n (%)	223 (26.1)	229 (21.9)	**.04**
Proportion[a] of agentic words, median [IQR]	0.0 [0.0-3.4]	0.0 [0.0-0.0]	**.03**
Gendered word(s) present in dictation, n (%)	231 (27.0)	246 (23.5)	.08
Proportion[a] of gendered words, median [IQR]	0.0 [0.0-4.0]	0.0 [0.0-0.0]	.07

[a]Proportions are relative to the number adjectives in the dictation.

Supplemental Digital Content 8. Number of gendered words divided by number of adjectives used in verbal dictations of operative performance feedback for female and male residents. Boxes represent interquartile ranges. Whiskers represent 1.5 times the 75th percentile value. All median values were 0. For cases by female attendings, gendered words represented less than 25% of all adjectives for both female and male residents, such that the distributions are not visible in the boxplot figure.

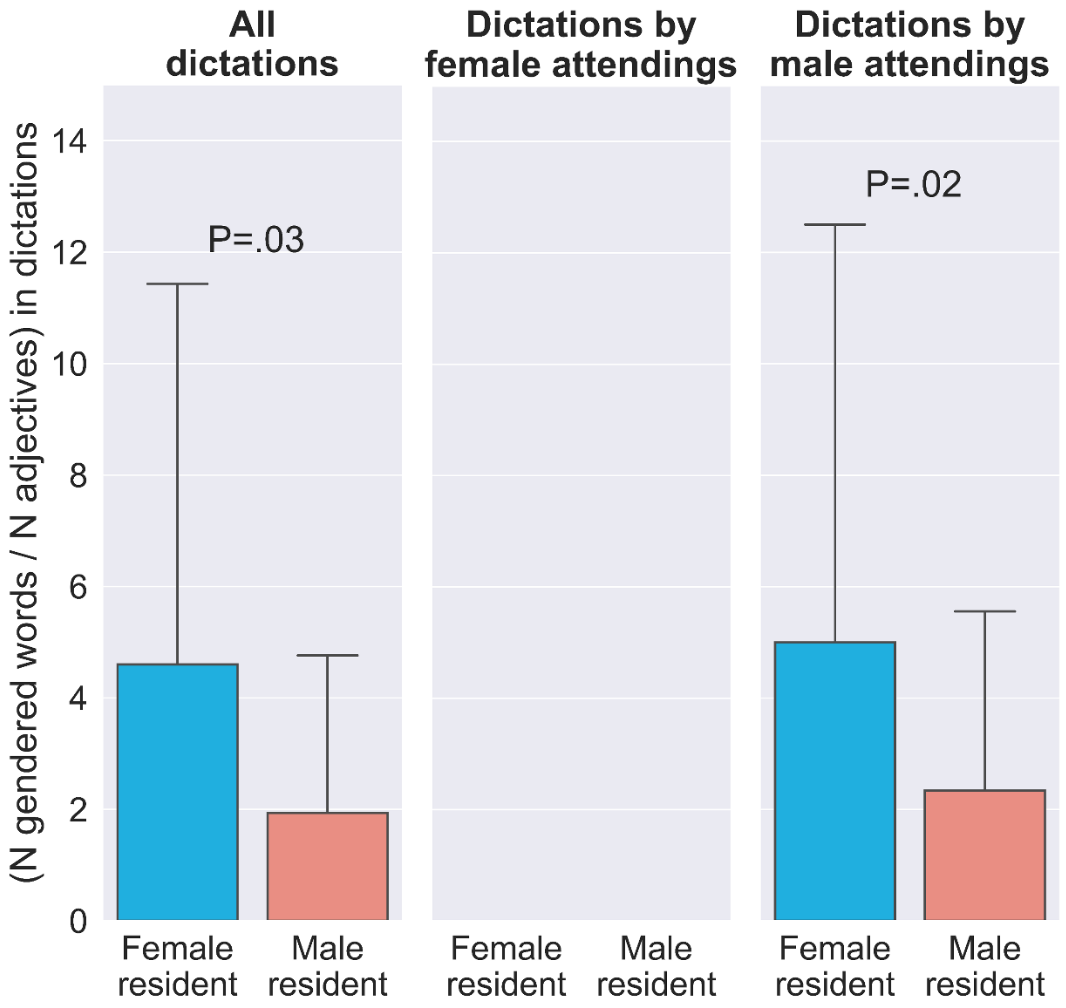

Supplemental Digital Content 9. Resident, attending, and case characteristics for cases in which the attending was male.

**Characteristics**, n (%)	**Female residents** (n=958)	**Male residents** (n=1,227)	**P**
**Resident postgraduate year**			
1	102 (10.6)	115 (9.4)	.35
2	125 (13.0)	153 (12.5)	.70
3	218 (22.8)	293 (23.9)	.54
4	223 (23.3)	310 (25.3)	.29
5	290 (30.3)	356 (29.0)	.54
**Total number of evaluations per resident**			
For the index case, median [IQR]	1.0 [1.0-2.0]	1.0 [1.0-2.0]	.67
For all cases, median [IQR]	16.0 [7.0-32.8]	18.0 [7.0-41.0]	**.009**
**Evaluation was initiated by the resident**	513 (53.5)	727 (59.3)	**.008**
**Top 20 most common procedures**			
Cholecystectomy (laparoscopic)	119 (12.4)	145 (11.8)	.69
Inguinal hernia repair (open)	98 (10.2)	113 (9.2)	.42
Appendectomy (laparoscopic)	67 (7.0)	70 (5.7)	.25
Ventral hernia repair (open)	43 (4.5)	37 (3.0)	.09
Inguinal hernia repair (laparoscopic)	22 (2.3)	36 (2.9)	.42
Cholecystectomy with IOC (laparoscopic)	27 (2.8)	27 (2.2)	.41
Exploratory laparotomy	19 (2.0)	28 (2.3)	.66
Ventral hernia repair (laparoscopic)	20 (2.1)	25 (2.0)	>.99
Umbilical hernia repair (open)	16 (1.7)	27 (2.2)	.44
Partial colectomy with anastomosis (laparoscopic)	11 (1.1)	14 (1.1)	>.99
Whipple	14 (1.5)	21 (1.7)	.73
Mastectomy, partial	11 (1.1)	26 (2.1)	.10
Small bowel resection with anastomosis (open)	16 (1.7)	13 (1.1)	.26
Hiatal hernia repair +/- fundoplication	4 (0.4)	23 (1.9)	**.003**
Colostomy/Ileostomy takedown (open)	7 (0.7)	11 (0.9)	.81
Renal transplant without recipient nephrectomy	8 (0.8)	15 (1.2)	.41
Breast biopsy or excision +/- needle localization	15 (1.6)	8 (0.7)	.06
Hepatectomy (open)	15 (1.6)	11 (0.9)	.17
Partial colectomy with anastomosis (open)	9 (0.9)	10 (0.8)	.82
Orthotopic liver transplant	11 (1.1)	13 (1.1)	.84
**Resident assessment of case complexity**			
Easiest 1/3	137 (14.3)	164 (13.4)	.53
Average	553 (57.7)	668 (54.4)	.13
Hardest 1/3	114 (11.9)	180 (14.7)	.07
Missing	154 (16.1)	215 (17.5)	.39
**Attending assessment of case complexity**			
Easiest 1/3	171 (17.8)	206 (16.8)	.53
Average	561 (58.6)	685 (55.8)	.21
Hardest 1/3	226 (23.6)	336 (27.4)	**.05**
Missing	0 (0.0)	0 (0.0)	>.99

Supplemental Digital Content 10. Resident, attending, and case characteristics for cases in which the attending was female.

**Characteristics**, n (%)	**Female residents** (n=208)	**Male residents** (n=290)	**P**
**Resident postgraduate year**			
1	21 (10.1)	17 (5.9)	.09
2	22 (10.6)	41 (14.1)	.28
3	70 (33.7)	82 (28.3)	.20
4	52 (25.0)	78 (26.9)	.68
5	43 (20.7)	72 (24.8)	.28
**Total number of evaluations per resident**			
For the index case, median [IQR]	1.0 [1.0-2.0]	1.0 [1.0-2.0]	.67
For all cases, median [IQR]	16.0 [6.0-32.2]	17.0 [7.0-35.8]	.28
**Evaluation was initiated by the resident**	147 (70.7)	191 (65.9)	.29
**Top 20 most common procedures**			
Cholecystectomy (laparoscopic)	18 (8.7)	34 (11.7)	.30
Inguinal hernia repair (open)	16 (7.7)	16 (5.5)	.36
Appendectomy (laparoscopic)	6 (2.9)	13 (4.5)	.48
Ventral hernia repair (open)	1 (0.5)	6 (2.1)	.25
Inguinal hernia repair (laparoscopic)	6 (2.9)	9 (3.1)	>.99
Cholecystectomy with IOC (laparoscopic)	2 (1.0)	2 (0.7)	>.99
Exploratory laparotomy	3 (1.4)	7 (2.4)	.53
Ventral hernia repair (laparoscopic)	3 (1.4)	6 (2.1)	.74
Umbilical hernia repair (open)	5 (2.4)	4 (1.4)	.50
Partial colectomy with anastomosis (laparoscopic)	7 (3.4)	14 (4.8)	.50
Whipple	4 (1.9)	2 (0.7)	.24
Mastectomy, partial	1 (0.5)	1 (0.3)	>.99
Small bowel resection with anastomosis (open)	2 (1.0)	1 (0.3)	.57
Hiatal hernia repair +/- fundoplication	2 (1.0)	2 (0.7)	>.99
Colostomy/Ileostomy takedown (open)	5 (2.4)	6 (2.1)	>.99
Renal transplant without recipient nephrectomy	3 (1.4)	2 (0.7)	.65
Breast biopsy or excision +/- needle localization	3 (1.4)	2 (0.7)	.65
Hepatectomy (open)	0 (0.0)	2 (0.7)	.51
Partial colectomy with anastomosis (open)	3 (1.4)	3 (1.0)	.70
Orthotopic liver transplant	0 (0.0)	0 (0.0)	>.99
**Resident assessment of case complexity**			
Easiest 1/3	26 (12.5)	28 (9.7)	.38
Average	133 (63.9)	181 (62.4)	.78
Hardest 1/3	22 (10.6)	47 (16.2)	.09
Missing	27 (13.0)	34 (11.7)	.68
**Attending assessment of case complexity**			
Easiest 1/3	28 (13.5)	46 (15.9)	.52
Average	129 (62.0)	169 (58.3)	.41
Hardest 1/3	51 (24.5)	75 (25.9)	.76
Missing	0 (0.0)	0 (0.0)	>.99

Supplemental Digital Content 11. Resident and attending assessments of resident operative performance and autonomy and the use of gendered words in dictated feedback for cases in which the attending was male.

**Evaluation results**, n (%)	**Female residents** (n=958)	**Male residents** (n=1,227)	**P**
**Resident assessment of resident performance**			
Critical deficiency	1 (0.1)	1 (0.1)	>.99
Inexperienced with procedure	133 (13.9)	158 (12.9)	.53
Intermediate	439 (45.8)	631 (51.4)	**.01**
Practice-ready	173 (18.1)	148 (12.1)	**<.001**
Exceptional	6 (0.6)	2 (0.2)	.15
Missing	206 (21.5)	287 (23.4)	.30
**Attending assessment of resident performance**			
Critical deficiency	2 (0.2)	0 (0.0)	.19
Inexperienced with procedure	112 (11.7)	205 (16.7)	**<.001**
Intermediate	509 (53.1)	647 (52.7)	.86
Practice-ready	304 (31.7)	342 (27.9)	**.05**
Exceptional	31 (3.2)	33 (2.7)	.52
Missing	0 (0.0)	0 (0.0)	>.99
**Resident assessment of resident autonomy**			
Show & tell	52 (5.4)	72 (5.9)	.71
Active help	434 (45.3)	582 (47.4)	.34
Passive help	224 (23.4)	292 (23.8)	.84
Supervision only	94 (9.8)	66 (5.4)	**<.001**
Missing	154 (16.1)	215 (17.5)	.39
**Attending assessment of resident autonomy**			
Show & tell	0 (0.0)	0 (0.0)	>.99
Active help	529 (55.2)	688 (56.1)	.70
Passive help	302 (31.5)	397 (32.4)	.71
Supervision only	127 (13.3)	142 (11.6)	.24
Missing	0 (0.0)	0 (0.0)	>.99
**Feedback from attendings to residents, per dictation**			
Sentiment score, median [IQR]	90.3 [3.4-99.5]	79.3 [2.2-99.3]	**.02**
Overall sentiment was positive, n (%)	566 (59.1)	685 (55.8)	.13
Number of words, median [IQR]	115.0 [64.0-191.0]	114.0 [65.0-201.5]	.45
Number of adjectives, median [IQR]	11.0 [7.0-18.0]	12.0 [7.0-19.0]	.29
Communal word(s) present in dictation, n (%)	19 (2.0)	30 (2.4)	.56
Proportion[a] of communal words, median [IQR]	0.0 [0.0-0.0]	0.0 [0.0-0.0]	.47
Agentic word(s) present in dictation, n (%)	277 (28.9)	290 (23.6)	**.006**
Proportion[a] of agentic words, median [IQR]	0.0 [0.0-4.2]	0.0 [0.0-0.0]	**.007**
Gendered word(s) present in dictation, n (%)	287 (30.0)	314 (25.6)	**.03**
Proportion[a] of gendered words, median [IQR]	0.0 [0.0-5.0]	0.0 [0.0-2.3]	**.02**

[a]Proportions are relative to the number adjectives in the dictation.

Supplemental Digital Content 12. Resident and attending assessments of resident operative performance and autonomy and the use of gendered words in dictated feedback for cases in which the attending was female.

**Evaluation results**, n (%)	**Female residents** (n=208)	**Male residents** (n=290)	**P**
**Resident assessment of resident performance**			
Critical deficiency	0 (0.0)	0 (0.0)	>.99
Inexperienced with procedure	28 (13.5)	25 (8.6)	.11
Intermediate	98 (47.1)	162 (55.9)	.06
Practice-ready	40 (19.2)	49 (16.9)	.55
Exceptional	0 (0.0)	0 (0.0)	>.99
Missing	42 (20.2)	54 (18.6)	.73
**Attending assessment of resident performance**			
Critical deficiency	0 (0.0)	0 (0.0)	>.99
Inexperienced with procedure	29 (13.9)	60 (20.7)	.06
Intermediate	112 (53.8)	131 (45.2)	.06
Practice-ready	60 (28.8)	86 (29.7)	.92
Exceptional	7 (3.4)	13 (4.5)	.65
Missing	0 (0.0)	0 (0.0)	>.99
**Resident assessment of resident autonomy**			
Show & tell	15 (7.2)	20 (6.9)	>.99
Active help	88 (42.3)	126 (43.4)	.85
Passive help	56 (26.9)	79 (27.2)	>.99
Supervision only	22 (10.6)	31 (10.7)	>.99
Missing	27 (13.0)	34 (11.7)	.68
**Attending assessment of resident autonomy**			
Show & tell	0 (0.0)	0 (0.0)	>.99
Active help	111 (53.4)	147 (50.7)	.59
Passive help	78 (37.5)	110 (37.9)	>.99
Supervision only	19 (9.1)	33 (11.4)	.46
Missing	0 (0.0)	0 (0.0)	>.99
**Feedback from attendings to residents, per dictation**			
Sentiment score, median [IQR]	99.3 [52.0-99.9]	96.1 [3.9-99.7]	**<.001**
Overall sentiment was positive, n (%)	156 (75.0)	182 (62.8)	**.005**
Number of words, median [IQR]	81.5 [49.8-138.0]	89.5 [56.2-153.0]	.10
Number of adjectives, median [IQR]	8.0 [5.0-13.0]	8.0 [5.0-13.0]	.37
Communal word(s) present in dictation, n (%)	1 (0.5)	7 (2.4)	.15
Proportion[a] of communal words, median [IQR]	0.0 [0.0-0.0]	0.0 [0.0-0.0]	.09
Agentic word(s) present in dictation, n (%)	46 (22.1)	62 (21.4)	.91
Proportion[a] of agentic words, median [IQR]	0.0 [0.0-0.0]	0.0 [0.0-0.0]	.67
Gendered word(s) present in dictation, n (%)	47 (22.6)	67 (23.1)	.91
Proportion[a] of gendered words, median [IQR]	0.0 [0.0-0.0]	0.0 [0.0-0.0]	.88

[a]Proportions are relative to the number adjectives in the dictation.
